# Would you like to leave Beijing, Shanghai, or Shenzhen? An empirical analysis of migration effect in China

**DOI:** 10.1371/journal.pone.0202030

**Published:** 2018-08-16

**Authors:** Tingting Liu, Hong Feng, Elizabeth Brandon

**Affiliations:** 1 Department of Applied Economics, School of Economics and Management, Beijing University of Technology, Beijing, China; 2 Department of Political Science, University of Chicago, Chicago, Illinois, United States of America; Ball State University, UNITED STATES

## Abstract

This study aims to estimate the migration effect of the overall samples and different flowing scales for the floating population from the perspective of personal wages. Although we used both the OLS and PSM methods to estimate the migration effect, we found that the PSM method was preferred in the study of migration as a result of the selection bias. The empirical results show that there is a significant difference in wage before and after migration. In fact, migration increased wages by 15.18% to 23.63% overall. Additionally, wages were increased by 44.96% to 59.20%, 23.06% to 26.18%, and 10.89% to 15.08% respectively for these three migration patterns: flowing into the three largest megacities, inter-provincial migration, and inter-city migration within a province, but for this pattern of inter-district migration within a city, the migration effect is not significant. We concluded that the floating population removing policies of the largest megacities maybe are effective because of the administrative power of their government. On the other hand, for these policies of non-largest megacities to attract labor and local employment and local urbanization near the floating population’s place of origin, they were not effective enough as a result of the lack of significant migration effect in these cities.

## Introduction

In China, the unbalanced development of the economy among provinces leads to a large wage gap in different regions. Generally, the developed largest megacities have a higher income level, which attracts more floating populations to these cities. Moreover, with the extensive construction of the traffic infrastructure in China, especially the high-speed railway, the act of migration is boomed by a strong internal motivation and increased convenience of the larger distance-scale “flowing”: that is, continuous movement back and forth between rural and urban areas and among different cities, and finally settling down in the original place of household registration. In 2015, about 247 million Chinese flowed, which accounted for 18% of the total population. In other words, there was one migrant in six persons. Meanwhile, about 48.8% of these migrants were less than 33 years old in 2013, while the proportion of the young migrants had increased to 51.1% in 2015 according to the 2016 Floating Population Report of China. It is suggested by statistical analysis that the migrants preferred to go to the developed largest megacities because they had more job opportunities and higher salaries, such as Beijing, Shanghai or Shenzhen. As more and more people flowed into the largest megacities, the “largest megacity disease”, which refers to varieties of drawbacks such as environmental pollution, traffic congestion and resource constraints caused by the excessive concentration of the urban population, industries, and transport and is defined in the China Green Development Index Report 2012: Regional Comparison, is becoming increasingly serious. Consequently, the government of the largest megacities raised the threshold to settle down and made some policies to remove the floating population (that is, the group who flowed from their original place of household registration to various places for a certain amount of time, while the household registration in their original place couldn’t transfer into the new residence city together with them as a result of the particular household registration policy in China.) (e.g. Beijing). In sharp contrast, some other cities always made some policies to attract the floating population, but the policy effects were not significant. The problem of orderly migration of the population has attracted more attention from the government. Meanwhile, the floating population is more carefully considering the choice of the new destination. Under these circumstances, the migration of China had become a unique and complicated economic phenomenon. The questions that have naturally arisen are why the population decided to flow, how to measure the migration effect, and whether or not the effect is significant, which also are the focuses of this paper. The studies about the migration in the last decades can be divided into two broad areas: one is mainly about the descriptive and statistical analysis, and the other is about the theoretical and empirical research.

The descriptive and statistical analysis mainly focuses on the determinants of migration and the willingness of the floating population to stay in the destinations. In previous studies, Barkley(1990) [1], Greenwood (1997) [2], Chiswick(1999) [3], Shen(2012) [4], Li, et al.(2002) [5], Chen, et al.(2002) [6], Polachek, et al.(1977) [7], Plane(1993) [8] and Zaiceva, et al.(2008) [9] found that the differences in wages, job opportunities, costs of living and public goods between the destination and original place are important determinants of a move. These studies also found that the migration propensity always changed over a person’s lifetime even if the personal variables remained the same, such as gender, marital status, education, etc. Ham, et al.(2005) [10] thought that the inter-regional difference in returns for the same skill plays an important role in the migration decision in the Federal Reserve Bank of New York Staff Reports. Harris, et al.(1970) [11] found that migration responds largely to regional disparities in prosperity. For the influencing factors on willingness to stay in the cities, Wang, et al.(2012) [12], Gao, et al.(2011) [13], Luo(2012) [14], Su, et al.(2005) [15], Wei(2013) [16] and Meng, et al.(2011) [17] all showed that the main factors were age, marital status, residential time in urban area, income level and public goods and services. The determinants of migration and the influencing factors on the willingness of the floating population to stay in the destinations are fundamental and crucial roles in the process of estimating the migration effect in our study.

In the empirical studies, there are some different perspectives to estimate the migration effect. Rozelle, et al.(1999) [18] estimated the migration effect on the agriculture productivity using the investigation data of farm households in Hebei and Liaoning provinces, which showed that the direct effect of migration was significant and negative on agricultural yields at least in the short run. Altonji, et al.(1991) [19] and Fields(1975) [20] studied the migration effect on the labor market of less-skilled and uneducated natives respectively, which found a negative effect on the job opportunities. Furthermore, the empirical study of Borjas(1985) [21] showed that it also had a negative effect on the earnings of the native-born in low skill occupations, but the increased labor supply due to migration was offset by higher demand for labor, so it presented a positive effect on native earnings overall. In a series of studies about the effect on natives’ earnings, Withers(1986) [22], Pope, et al.(1993) [23], Addison, et al.(2002) [24], Butcher, et al.(1991) [25], Greenwood, et al.(1986) [26] and Borjas(1993) [27] all found that migration had a limited effect or no negative effect on natives’ earnings.

From the perspective of migrants themselves, most of the migration theoretical models usually treat migration as an investment in human capital. In fact, the decision to change regions alters the rate of return to human capital investment in substance, so a lot of empirical research estimates the change degree in wage and earning before and after migration. Most migration models clearly predict a higher wage for those who flow into the new destinations, but the literature actually has not reached a consensus on the wage change. In studies on this topic, negative, zero, and positive results were all reported. Shaw(1991) [28] predicted that the wage changes were positive from the three perspectives of change region, change industry, and change region and industry simultaneously. The positive growth in wage also was predicted by Bartel(1979) [29], Hunt, et al.(1985) [30], Gabriel, et al.(1995) [31] and Yankow(2003) [32] from different perspectives of migration. The negative effect on wage was found by Tunali(2000) [33] for a substantial portion of migrants, while only a small minority got a higher wage than before which made migration essentially a lottery. Some studies found that the wage change was statistically insignificant for some types of migrants, such as Bartel(1979) [29] for older migrants, Hunt, et al.(1985) [30] for one-time migrants because of high information cost, and Yankow(2003) [32] for migrants with Master and Doctor degrees.

Most of the empirical studies on the wage changes before and after migration are about migrants of the USA, which include the internal and international migration. However, the migration effect on wage is relatively rare worldwide. A major problem, like what Stark, et al.(1985) [34] said, was the absence of variables in the dataset, because this kind of study needs the survey dataset to provide information on individual wages at their residential location and original region, their migrant or non-migrant status, individual characteristics (for example, age, education, and marital status) and so on. Additionally, it needs a large sample size to get accurate results. So far, there are not many empirical studies about the migration effect of floating populations on wage changes before and after migration, especially for the unique phenomenon of floating populations of China. However, the direct effect of migration that is caused by the big wage gap among the regions is the wage change after the migrants moved. Hence, this paper will use the scientific statistical method and representative dataset to do an analysis about the migration effect of floating populations of China from the perspective of wage change on the two following aspects: 1. Estimate the migration effect of the overall and different flowing scales for floating populations. The different flowing scales include the four migration patterns: flowing into the three largest megacities: Beijing, Shanghai, and Shenzhen, which are the representational cities of the three developed economic circles of China: Beijing-Tianjin-Hebei Metropolitan Region, the Yangtze River Delta, and the Pearl River Delta respectively, inter-provincial migration (not including the floating population who come from other provinces flow into the three largest megacities), inter-city migration within a province, and inter-district migration within a city. 2. Contrast and analyze the migration effects of the four migration patterns, and discuss methods to improve the effect of the removing and attraction policies for floating populations.

The paper is organized as follows: review the relative theories of migration and introduce the analysis method of the paper; introduce the econometric model and estimate the migration effect of the overall and different flowing scales for floating population respectively using the OLS and PSM; and conclude the paper.

## Related theories and analytical methods

### The dualistic economic structure theory and Pareto improvement theory of migration

The economist Lewis(1954) [35] proposed the dualistic economic structure theory, which is that there are two sectors in the closed economy: one is the urban sector with industry and services, and the other is the agricultural sector which is dominated by the agriculture and handicraft industries. Additionally, under the assumption of an unlimited supply of labor, since wages of the urban sector are higher than that of the agricultural sector, if farmers who work in agriculture are not constrained, they will move into cities to find jobs. Differences in return to labor play an important role in the migration decision. In the realistic economy of China, there is the same situation as described above: labor from agriculture flows into the cities for higher income. At the same time, there is also a situation that urban labor flows among different cities to find jobs that meet their requirements. It shows that the surplus labor force exists not only in the agricultural sector but also in the urban sector. Specifically, the surplus labor force that exists in the agricultural sector and the urban sector and flows into the new destinations is the floating population who will be studied in this paper. The unbalanced development of the economy and the sharp difference of wage among regions contribute to the migration phenomenon of the surplus labor force in China.

The phenomenon of population migration is very beneficial to the individuals, the families, the enterprises, and the economic development of society as a whole. From the view of individuals, the floating population can easily find jobs that meet their requirements and reach their expectations by expanding the scale of employment. From the angle of enterprises, they can easily employ the workers that meet the requirements of the job, and it is also possible to reduce the labor cost of the enterprise. In terms of the overall economy of China, the floating population can significantly increase national productivity and improve labor efficiency. Overall, population migration essentially is a reallocation of human resources, which is a Pareto improvement for each side. As the elderly population increases in China and many developed countries, migration becomes very important for the redistribution of the labor force and is necessary for economic development.

### The ordinary least squares (OLS) regression and the propensity score matching (PSM) method

In this study, the analysis of the migration effect will focus on calculating or estimating the wage change after migration. There are two methods used to estimate it: one is the ordinary least squares (OLS) regression, and the other is the propensity score matching (PSM) method.

#### The ordinary least squares (OLS) regression

The OLS is a common method for estimating the relationship between a dependent variable and a set of independent variables by a linear function. In order to get the optimal linear unbiased estimators, it needs to meet several requirements, including that the regressors are exogenous and the errors are homoscedastic and serially uncorrelated.

For the linear model of OLS in this study, the dependent variable is the log of the average monthly wage of every surveyed sample. Migration or non-migration as a dummy variable is put into the regression function. A series of control variables are added to ensure that the model is reliable. The migration effect in wage is reported by the regression coefficient of the dummy variable. The OLS method in this problem can only roughly estimate the relationship between the status of migration or non-migration and wage. In fact, it is difficult to explore the direct effect between them, because the dummy variable migration or non-migration is a choice variable and is not randomly assigned for all population. The decision of migration or non-migration is related to the control variables of a migrant. In other words, the choice will be influenced by the other independent variables and will not be random, so the selection bias will appear in the regression results of OLS, biasing the migration effect. Nakosteen, et al.(1980, 1982) [36, 37] were the first to offer evidence for self-selection of migration. Gabriel, et al.(1995) [31] and Robinson, et al.(1982) [38] also found self-selection of migration in their studies.

#### The propensity score matching (PSM) method

Selection bias is a critical problem in the study of migration effect. In order to make the migration effect as accurate as possible, it is necessary to examine whether migration is a random decision or not. Rosenbaum, et al.(1983) [39] addressed this problem using the propensity score matching (PSM) method. The PSM concentrates on the randomness of a migration decision and aims to estimate an unconditional migration effect. Meanwhile, it transfers the multi-dimensional control variables problem, which is the impracticality of matching so many variables in the empirical analysis, into a one-dimensional problem using the new concept of propensity values (also called propensity scores) of the control variables. In the process of matching, the propensity scores, which are the migration probabilities conditional on X, are estimated by the Logistic or Probit regression model, where,

$$\begin{array}{c} P(X)=Pr(M=1|X). \end{array}$$ (1)

The PSM focuses on the propensity values rather than the specific values of control variables. It is a new way to concentrate on the control variables and determine the causal relationship only when the control variables meet specific requirements. This is considered a matching of control variables. In the matching process of the control variables, we obtain the causal relationship accurately between the dependent variable and the explanatory variable. In the PSM method, both the explanatory variable and the control variables are taken into account [39]. In other words, regardless of the number of control variables, we obtain a relatively accurately causal relationship using the PSM. Therefore, from the point of view of matching, the PSM is an effective method to get an accurate causal relationship between the dependent variable and the explanatory variable in the situation of multiple control variables. Most importantly, the PSM is given more attention and constantly improved, so it gets causal inference through more rigorous statistical techniques [40–42]. In order to avoid the problem of selection bias and obtain accurate results, this study will attempt to estimate the migration effect of the floating population in wage using the PSM method.

From the statistical point of view, the PSM introduces the counterfactual theories of causation. We can explain the counterfactual process using the specific example of the migration effect of floating populations. The observable fact is that someone flows to a new destination and gets a higher salary. The counterfactual situation refers to the impossible scenario that the same migrant simultaneously works in his original job location, receiving a lower salary. Hence, this counterfactual situation could not be observed. The causal relationship between wage and status of migration or non-migration refers to the average value of the wage differences between new and old locations for the same group in a statistical sense. In other words, the causal relationship in statistics is the average difference between the facts that could be observed and the counterfactual situation that could not be observed. In the following equation, the variable wage denotes the wages of the population, where wage_1_ is the wage of flowing, and wage_0_ is the wage of non-flowing. M is the dummy variable, where M = 1 denotes migration, and M = 0 denotes non-migration. X denotes the vector of control variables, such as gender, education, etc. The unbiased estimation calculated by the PSM is E(wage_1_|X, M = 1)—E(wage_0_|X, M = 1), which is the Average Treatment effects on the Treated (ATT), so the ATT = E(wage_1_|X, M = 1)—E(wage_0_|X, M = 1) shows the average wage difference between the migrants and the same group if they don’t flow. The ATT denotes the direct effect of the causal relationship.

As mentioned above, the migration effect estimated by the OLS method is biased, because the estimated result of the OLS is E(wage_1_|X, M = 1)—E(wage_0_|X, M = 0), so the selection bias is [E(wage_1_|X, M = 1)—E(wage_0_|X, M = 0)]—[E(wage_1_|X, M = 1)—E(wage_0_|X, M = 1)] = E(wage_0_|X, M = 1)—E(wage_0_|X, M = 0), which is calculated from the results of OLS and PSM. As can be seen from the formula, the selection bias refers to the difference between the wages of the population that work in the new cities if they were to still work in the original place and the wages of the population that actually work in the original place. The selection bias makes the result of the OLS regression biased.

In the formula above, E(wage_1_|X, M = 1) and E(wage_0_|X, M = 0) that could be observed are the facts, and E(wage_0_|X, M = 1) that could not be observed is the counterfactual situation. In real life, we could observe the migration or non-migration for the same group, but it is impossible to observe the migration and non-migration for the same group simultaneously. Therefore, the PSM creates a new way to estimate the counterfactual situation.

In general, the steps using the PSM to estimate the causal relationship [43, 44] are below: (1) Predicting the propensity scores: using the Logit or Probit model to calculate the migration probability: P(M = 1|X); (2) Matching based on the propensity scores: matching samples by the methods of the nearest neighbor matching, radius matching, kernel matching, etc; (3) Estimating the coefficient of the explanatory variable based on the matching samples: getting the wage difference one by one between a migrant and a non-migrant who have the same propensity scores, and calculating the average value of the differences, which is the ATT denoting the migration effect in wage; (4) Balancing test: after getting the ATT using the PSM, it is necessary to do the balancing test for the control variables in order to ensure the high quality of matching and the accuracy of estimation.

## Modeling and results

### The result of data statistics

The primary data source in this empirical research is the Migrants Population Dynamic Monitoring Survey Data of the National Health and Family Planning Commission of China (NHFPCC) in 2015. The survey samples include 206,000 migrants and 16,000 non-migrants of 332 cities in China.

This dataset comes from the official survey with good national representation ideally suited for studying migration using the PSM. It is characterized by the scientific sampling method and a large sample size, so the empirical analysis using the dataset can better reflect the relevant problems of the national floating population and non-floating population. Additionally, the dataset of the NHFPCC in 2015 provides a relatively rich array of variables which includes every sample’s age, gender, education, original region, destination, and so on. Thus, it is possible to match the characteristic variables of the samples, estimate the migration effects of different flowing scales, and further compare the migration effects of different flowing scales.

In this study, we make some selection criteria for the data of the floating population and non-floating population in order to make the samples suitable for this research. First, the age of the samples is limited from 18 to 65 years old to ensure that the wages come from their jobs rather than from retirement funds. Second, in order to avoid the estimation bias that is caused by investigation data, we deleted the samples in which the wages are less than 1000RMB. The national minimum wage in China was more than 1000RMB/month in 2015, so the wages that are less than 1000RMB likely contain statistical errors. Finally, the number of effective samples is about 180,200, which includes a floating population of 171,400 and a non-floating population of 8,800.

In this paper, the PSM is used to estimate the migration effect by matching the control variables of the floating population and non-floating population. However, there is a large difference in the sample size for the two groups, which will result in an insufficient data utilization rate and matching difficulty in the matching process. Thus, we extracted samples from the floating population at 3.5 times the number of non-floating population samples randomly. Moreover, the extracted samples also meet the balanced distribution of the original place of the floating population. Finally, 30,365 samples of the floating population were extracted. We need to notice that, in order to meet the balanced distribution of the original place, we extracted the samples from each province, but the sample number of the floating population in some provinces is less than 3.5 times the number of non-floating population, so the total extracted samples of floating population are less than the 3.5 times the total number of non-floating population. We performed the study using the extracted samples of the floating population and the 8,800 samples of the non-floating population.

The basic characteristics (descriptive statistics) of the 39,165 samples are listed in Table 1. The third and fourth columns provide the proportion of each category for each variable of the floating population and non-floating population respectively.

**Table 1:** The basic characteristics of the samples.

Variable	Indicators	Floating population	Non-floating population
*Gender*	Male	58.42%	54.53%
	Female	41.58%	45.47%
*Education*	Never went to school	1.43%	0%
	Primary school	12.44%	2.30%
	Junior high school	51.04%	19.02%
	High school/Secondary school	22.07%	25.74%
	Specialist	8.33%	23.31%
	Undergraduate and graduate	4.69%	29.63%
*Marital status*	Unmarried	19.36%	19.80%
	First marriage	76.90%	74.59%
	Remarriage	1.38%	1.79%
	Divorce and widowhood	2.36%	3.82%
*Household registration type*: *countryside or city*	Countryside	84.15%	4.56%
	City	15.85%	95.44%
*Income* (*Unit*: *RMB*)	Less than 2000	18.19%	19.44%
	2000-4000	52.02%	45.72%
	4000-6000	19.27%	20.38%
	6000-8000	5.19%	6.18%
	8000-10000	2.91%	4.91%
	10000-50000	2.36%	3.37%
	More than 50000	0.06%	0%

It can be seen from the last two columns of Table 1 that there is a little difference in the proportions of gender, marital status and income between the floating population and the non-floating population. However, the proportions of education and household registration type are quite different. The specific description and explanations are as follows:

- Gender and marital status: For these two variables, the proportions of each category are not significantly different between the floating population and the non-floating population. Specifically, the proportions of gender are relatively balanced in each group, while the marriage rate, including first marriage and remarriage, is close to 80% respectively in both the floating population and non-floating population.
- Education: For the floating population, a majority of people have only a primary, junior high, and senior high school level of education. These people are generally more hard-working, and most of them have specialized professional skills, which make them able to find jobs more easily in the new cities and earn relatively higher wages than those of their original places. On the other hand, a majority of the non-floating population has an undergraduate or graduate education because a higher education enables them to more easily find a stable job in their original places. Thus, this population is less willing to flow.
- Household registration type: In the floating population, a substantial portion, nearly 85%, came from the countryside. This rural population aspires to work in non-agricultural sectors, but these opportunities in rural areas are very scarce. Accordingly, these people seek jobs elsewhere. In the non-floating population, the proportion of the urban residents is relatively large, which indicates that there are relatively more employment opportunities in the cities.
  By considering both the statistical results of household registration type and marital status for the floating population, we infer that many couples from the countryside work in the cities together. While this inevitably leads to children and older people who are left behind, these couples consider the sacrifice worth it in order to obtain a higher income and improve the quality of life for their loved ones.
  In terms of relating the education level and the household registration type of the floating population, we know that about half only has a junior high school level of education, while most of the floating population comes from the countryside. This observation indicates that the floating population coming from rural settings is a major source of manual labor in the cities and that this kind of manual labor can more easily to find a job and earn high wages in the cities.
- Income: As can be seen from Table 1, the percentage of the population for every interval of wage is not significantly different between the floating population and the non-floating population. However, it cannot be concluded that the migration effect is insignificant because the heterogeneity among the people is not considered. Thus, it is critical to pay attention to the heterogeneity of population in research of the migration effect.

Table 1 presents the statistical results of the floating population and non-floating population samples. From the analysis above, we found that it was necessary to estimate the migration effect on wage change before and after migration, which essentially reflects the degree of improving quality of life for the floating population. Meanwhile, in the process of estimating the migration effect, there is maybe a serious selection bias that must be addressed in order to correctly obtain the degree of wage growth after migration. In order to determine the impact of selection bias, the study will use the two methods, OLS and PSM, to estimate the migration effect simultaneously.

### The estimation result of the OLS for the migration effect of the overall floating population

The estimation of the migration effect of the floating population using the OLS method is based on the following regression equation:

$$\begin{array}{c} \text{ln}\left(\text{wage}\right)=\beta_{0}+\beta_{1}M+\beta_{2}X+u. \end{array}$$ (2)

In the equation above, ln(wage) is the log of the average monthly wage for every sample; *β*_0_ is the constant term; M is a binary dummy variable (M = 1 if an individual flows and M = 0 otherwise), and the coefficient *β*_1_ reports the increased percentage of monthly wage of the floating population compared to the non-floating population, which is the migration effect; X is a vector of control variables that will directly affect wage and also affect the migration decision to some extent; *β*_2_ represents the coefficient vector on X; and u is the residuals. In the paper, the vector of control variables, X, includes gender, household registration type, marital status, junior high school or not, original regions (The original regions of the samples are divided into eastern, western, central and three northeastern provinces of China. The three northeastern provinces was a net outflow region over the past 10 years, so it will be discussed separately as a group.), and whether or not the education funding from the government is more than 40000RMB/student/year (the 2015 median education funding across all provinces) in the original place. The result of the migration effect in monthly wages estimated by the OLS method is shown in Table 2. It can be seen that migration could increase wages by 17.21% with a 1% significance level.

**Table 2:** The migration effect on the monthly wage-OLS.

Variable	Coefficient	Statistics	Value
*M*	0.1721***^1^	*R*-*squared*	0.0982
		*Adj R-squared*	0.098
		*F*-*value*	473.68
*Other* ^2^	Skip over	*P*-*value*	0
		*Number of obs*	180700

^1^ *** means significant at 1% level.

^2^ Other variables include gender, household registration type, marital status, junior high school or not, original regions, and whether or not the education funding from the government is more than 40000RMB/student/year in the original place.

### The estimation result of the PSM for the migration effect of the overall floating population

As mentioned above, the migration effect which is estimated by the OLS is not entirely accurate as a result of the selection bias, so we will use the PSM which addresses the selection bias to estimate the net effect of migration from the perspective of wage. Additionally, the one-to-one nearest neighbor matching (with replacement), which is the simplest matching method in the PSM, will be used to estimate the effect.

#### The probability of migration (the propensity score)

We estimated the probability of migration using the binary logit model. The regression equation is as follows:

$$\begin{array}{c} M=\beta_{0}+\beta_{1}X+u. \end{array}$$ (3)

In the equation notation above, M, *β*_0_, X and u denote the same variables as for the OLS. In theory, the vector of control variables, X, should include the variables that meet the following requirements: they affect whether the population decides to migrate; they are pretreatment variables that will not be affected by the decision of migration or non-migration; and they are time-invariant individual characteristic variables. In the equation above, X includes the same control variables as in the OLS model. *β*_1_ represents the coefficient vector on X.

We put the Migrants Population Dynamic Monitoring Survey Data mentioned above into the logit model. The results showed that females are slightly less likely to migrate than males; people from the countryside are much more likely than those from the cities; singles are slightly higher than married people. With the central region as a benchmark, the migration probability for the eastern region is much lower, while it is much higher in the western region and slightly higher in the three northeastern provinces. People who finished junior high school are slightly more likely to migrate than those who didn’t, and migration from regions with more than 40000RMB/student/year in education funding is much lower than from other regions. From the above analysis, we see that the countryside population is significantly willing to migrate, and that increased government funding for education will contribute to the long-term survival of the population.

#### Estimate the ATT

After getting the propensity scores, the Average Treatment effects on the Treated (ATT) which is the migration effect could be estimated by

$$\begin{array}{c} \hat{\text{ATT}}=\frac{1}{N_{1}}\sum {}_{i:M_{i}=1}\left(ln{\left(wage\right)}_{i}-\hat{ln{\left(wage\right)}_{0i}}\right). \end{array}$$ (4)

This equation only denotes the treatment group. N_1_ = ∑_i_M_i_ is the number of the migrants in the common support range, and $\sum {}_{i:M_{i}=1}\left(\text{ln}{\left(\text{wage}\right)}_{i}-\hat{\text{ln}{\left(\text{wage}\right)}_{0i}}\right)$ is the sum of the differences of the log of each person’s monthly wage in their new city and the wage they would have made if they had continued working in the original place. The $\hat{\text{ln}{\left(\text{wage}\right)}_{0i}}$ value from the counterfactual situation could be calculated from a non-migrant with the same propensity score as the migrant. Similarly, the Average Treatment effects on the Untreated (ATU) can be estimated by

$$\begin{array}{c} \hat{\text{ATU}}=\frac{1}{N_{0}}\sum {}_{j:M_{j}=0}\left(\hat{ln{\left(wage\right)}_{1j}}-ln{\left(wage\right)}_{j}\right). \end{array}$$ (5)

This equation only denotes the untreated group. N_0_ = ∑_j_(1 − M_j_) is the number of the non-migrants in the common support range, and $\sum {}_{j:M_{j}=0}\left(\hat{\text{ln}{\left(\text{wage}\right)}_{1j}}-\text{ln}{\left(\text{wage}\right)}_{j}\right)$ is the sum of the differences of the log of each person’s monthly wage if they had moved to a new city and their actual wage. We could get the value of the $\hat{\text{ln}{\left(\text{wage}\right)}_{1j}}$ in the same way as the $\hat{\text{ln}{\left(\text{wage}\right)}_{0i}}$. Similarly, the Average Treatment Effects (ATE) on the whole sample can be estimated by

$$\begin{array}{c} \hat{\text{ATE}}=\frac{1}{N}\sum {}_{i=1}^{N}\left(\hat{ln{\left(wage\right)}_{1i}}-\hat{ln{\left(wage\right)}_{0i}}\right). \end{array}$$ (6)

In the equation above, N = N_0_ + N_1_ is the number of the total samples in the common support range; if M_*i*_ = 1, then $\hat{\text{ln}{\left(\text{wage}\right)}_{1i}}=\text{ln}{\left(\text{wage}\right)}_{i}$; similarly, if M_*i*_ = 0, then $\hat{\text{ln}{\left(\text{wage}\right)}_{0i}}=\text{ln}{\left(\text{wage}\right)}_{i}$. Thus, $\sum {}_{i=1}^{N}\left(\hat{\text{ln}{\left(\text{wage}\right)}_{1i}}-\hat{\text{ln}{\left(\text{wage}\right)}_{0i}}\right)$ is the sum of the differences between the fact and the counterfactual situations for the two groups.

The 39,165 samples, including 30,365 floating population samples that were extracted randomly and meet the balanced distribution of the original place and 8,800 non-floating population samples from the 2015 NHFPCC dataset, were put into Stata using one-to-one nearest neighbor matching (with replacement) of the PSM. The results are shown in Tables 3 and 4.

**Table 3:** The results of one-to-one nearest neighbor matching (with replacement).

Independent variable	Sample	Treated	Untreated	Difference	S.E.	t-stat
*ln*(*wage*)	Unmatched	8.1489	8.1922	-0.0433	0.0066	-6.58
	ATT	8.1489	7.9891	0.1598	0.0571	2.8
	ATU	8.1922	8.3768	0.1846		
	ATE			0.1654		

**Table 4:** Common support range of one-to-one nearest neighbor matching (with replacement).

Treatment assignment	Off support	On support	Total
*Untreated*	0	8761	8761
*Treated*	0	30404	30404
*Total*	0	39165	39165

The fifth and seventh columns of Table 3 present the results of matching estimation and t-statistics of the migration effect in wage change using the same model as OLS. The value of ATT is about 15.98%, which is the migration effect. Meanwhile, the T-value of 2.8 is bigger than the boundary value of 1.96, so the result is significant. In other words, the floating and non-floating populations with the same propensity scores have a significant difference in the monthly wage of about 15.98%, which means that migration could increase monthly wages by nearly 16%. From Table 4, we note that all 39,165 samples of the treatment and untreated groups are within the common support range.

#### Balancing test

In the process of estimating the ATT using the PSM, some assumptions must hold. The fundamental assumption, first studied by Rosenbaum, et al.(1983) [39], is called the Ignorable Treatment Assignment (ITA). In the context of the migration effect, it means that (ln(wage)_0*i*_, ln(wage)_1*i*_) and M_*i*_ are independent when the control variables X are given,

$$\begin{array}{c} (\text{ln}{\left(\text{wage}\right)}_{0i},\text{ln}{\left(\text{wage}\right)}_{1i})⊥M|X. \end{array}$$ (7)

If the ITA assumption is satisfied, then

$$\begin{array}{c} \left(\text{ln}{\left(\text{wage}\right)}_{0i},\text{ln}{\left(\text{wage}\right)}_{1i}\right)⊥M\left|X\Rightarrow \left(\text{ln}{\left(\text{wage}\right)}_{0i},\text{ln}{\left(\text{wage}\right)}_{1i}\right)⊥M\right|P\left(X\right). \end{array}$$ (8)

Most importantly, (ln(wage)_0i_, ln(wage)_1i_) ⊥ M|P(X) is implied by the condition that M ⊥ X|P(X). In general, it is difficult to directly test the ITA assumption, (ln(wage)_0i_, ln(wage)_1i_) ⊥ M|X, so we instead check if the variables are balanced: that is, whether or not M ⊥ X|P(X).

In this research, we need to test whether or not the decision of migration or non-migration is made randomly for individuals who have the same propensity scores. In other words, we have to check whether or not the floating and non-floating populations with the same propensity score have the same individual characteristics X. For the estimation above, the results of this balancing test are shown in Tables 5 and 6.

**Table 5:** The results of the balancing test-1.

Variable	Unmatched Matched	Mean		%bias	%reduce |bias|	t-test	
		Treated	Control			t	p>|t|
*Gender*	U	0.5869	0.5443	8.60		7.11	0.00
	M	0.5869	0.5887	-0.40	95.70	-0.45	0.65
*Household registration type*: *countryside or city*	U	0.1769	0.9550	-253.40		-183.23	0.00
	M	0.1769	0.1769	0.00	100.00	0.00	1.00
*Marital status*	U	0.7781	0.7633	3.50		2.93	0.00
	M	0.7718	0.7802	-0.50	86.30	-0.61	0.54
***Eastern of China***	U	0.2361	0.6141	-82.80		-70.96	0.00
	M	0.2361	0.2361	0.00	100.00	0.00	1.00
***Western of China***	U	0.3977	0.1186	67.30		50.30	0.00
	M	0.3977	0.3977	0.00	100.00	0.00	1.00
***Three northeastern provinces of China***	U	0.0995	0.1146	-4.90		-4.09	0.00
	M	0.0995	0.0992	0.10	97.60	0.15	0.88
*Junior high school or not*	U	0.8805	0.8820	-0.50		-0.38	0.70
	M	0.8805	0.8829	-0.70	-60.00	-0.92	0.36
*Education funding* ^1^	U	0.0047	0.2001	-68.10		-81.18	0.00
	M	0.0047	0.0047	0.00	100.00	0.00	1.00

Note: The bolder and italic ones mean that the central region is the benchmark in the variables of the original regions.

^1^ The education funding means whether or not financial support from the government is more than 40000RMB/year/student in the original place.

**Table 6:** The results of the balancing test-2.

Unmatched Matched	Ps R^2^	LR *χ*^2^	p> *χ*^2^	MeanBias	MedBias	B	R	%Var
*U*	0.523	2177.7	0.0	61.1	37.9	257.5	1.55	88
*M*	0.0	1.43	0.994	0.2	0.1	1.0	1.03	0.0

Table 5 shows the results of balancing test for the matched and unmatched simultaneously. For matched samples, the balancing test calculates the mean of every variable for the floating population sample and its closest non-floating match, and then finds the mean bias between treatment and untreated groups. For unmatched samples, the balancing test simply calculates the mean of each variable and the mean bias for the floating and non-floating populations without any matching. Finally, we calculate the percentages of bias reduction. The full results of the test can be seen in Table 5, but we note the following. First, each variable showed a large difference in means before matching, but no variables showed a significant difference after matching. Second, the mean bias of each variable fell more than 85% after matching. Additionally, the t-tests and p-values show that the control variables are well balanced after matching. The p-value of each independent variable in Table 5 is very large after matching, so we cannot reject the null hypothesis that the variables are identically distributed. In summary, while the distributions of each independent variable differ before matching, there is no significant difference in the distributions after matching between floating population and non-floating population.

As shown in Table 6, before matching, the value of Pseudo R^2^ was big, and the P-value was small, but after matching, the value of Pseudo R^2^ became small and the P-value became big. So for the overall variables, the distribution differs before matching, but there is no significant difference in the distribution after matching. Therefore, it is very necessary to use PSM to estimate the migration effect.

All test results showed that the control variables have good matching and are therefore well balanced after matching.

#### Estimate the ATTs using various matching methods and some sensible parameter values in the PSM

In the PSM estimation process, there are various common matching methods, such as k-nearest neighbor matching(k = 1,2,3…), caliper matching(k-nearest neighbor matching of different caliper widths), caliper and radius matching, kernel matching, local linear regression matching, Mahalanobis metric matching, etc. Each method has advantages and disadvantages, and there is no definite conclusion on which matching method and parameter values (k, caliper widths, matching methods with or without replacement, etc.) should be chosen for a specific problem. Thus, this paper uses several sets of matching methods and parameter values to estimate the migration effect and compares the results. The ATTs which are estimated using different matching methods and parameter values are shown in Table 7.

**Table 7:** The migration effect on the average monthly wage for the different matching methods and parameter values.

Matching methods	Sample		Parameters	ATT	t-value
	untreated	treated			
*k-nearest neighbor matching*	8761	30404	k = 1	0.1598	2.80
	8761	30404	k = 2	0.1741	2.81
	8761	30404	k = 4	0.1538	2.63
	8761	30404	k = 7	0.1673	3.35
	8761	30404	k = 12	0.1713	3.53
*Caliper matching*: *k-nearest neighbor matching of different caliper widths*	8761	30404	& = 0.001,k = 1	0.1599	2.80
	8761	30404	& = 0.005,k = 1	0.1599	2.80
	8761	30404	& = 0.1,k = 1	0.1598	2.80
	8761	30404	& = 0.001,k = 2	0.1740	2.81
	8761	30404	& = 0.005,k = 2	0.1742	2.81
	8761	30404	& = 0.1,k = 2	0.1741	2.81
	8761	30404	& = 0.001,k = 7	0.1669	3.34
	8761	30404	& = 0.005,k = 7	0.1676	3.36
	8761	30404	& = 0.1,k = 7	0.1673	3.35
	8761	30404	& = 0.001,k = 12	0.1733	3.60
	8761	30404	& = 0.005,k = 12	0.1714	3.53
	8761	30404	& = 0.1,k = 12	0.1712	3.53
*Caliper and radius matching*	8760	30397	& = 0.001	0.1880	4.12
	8760	30397	& = 0.005	0.1956	4.63
	8761	30397	& = 0.01	0.1935	6.25
	8761	30404	& = 0.1	0.1934	3.84
*Kernel matching*	8761	30404	k(epan).bw(0.06)	0.1932	3.95
	8761	30404	k(epan).bw(0.03)	0.1674	5.01
	8761	30404	k(epan).bw(0.01)	0.1998	6.12
	8761	30404	k(normal).bw(0.06)	0.1934	3.99
	8761	30404	k(normal).bw(0.03)	0.1518	4.12
	8761	30404	k(normal).bw(0.01)	0.1678	6.07
*Local linear regression matching*	8761	30404	k(epan).bw(0.06)	0.2333	4.09
	8761	30404	k(epan).bw(0.03)	0.2363	4.14
	8761	30404	k(epan).bw(0.01)	0.1924	3.37
	8761	30404	k(normal).bw(0.06)	0.2222	6.65
	8761	30404	k(normal).bw(0.03)	0.2359	6.81
	8761	30404	k(normal).bw(0.01)	0.2213	5.98
*Mahalanobis metric matching*	8761	30404	m = k = 1	0.1800	1.97
	8761	30404	m = k = 2	0.1569	1.99
	8761	30404	m = k = 5	0.1526	2.41
	8761	30404	m = k = 7	0.1524	2.81

From Table 7, we see that the ATT values are roughly the same for each set of matching method and parameter values, so the empirical results are sufficiently robust. In particular, the t-values of ATTs are all bigger than the boundary value of 1.96, so we should reject the null hypothesis that there is no wage difference between the floating and non-floating populations. From Table 7, we see the wages of the floating population in China tend to be 15.18% to 23.63% higher overall than those of their non-floating counterparts. This implies that the migration can increase wages by 15.18% to 23.63%, while the OLS estimated a migration effect of only 17%. Thus, there is a difference of estimation between the two methods. This also provides some evidence of a selection bias of migration, although it is not very serious for the overall national samples.

### Which kind of migration is the most effective?

#### The migration effects of the four different flowing scales: Overall sample

The migration effect may depend on the scale of migration. In order to facilitate the discussion, the floating population dataset of the 2015 NHFPCC was divided into four migration patterns depending on the scale: flowing into the three largest megacities, inter-provincial migration, inter-city migration within a province, and inter-district migration within a city. For each migration pattern, we randomly extracted samples of the floating population at 3.5 times the number of non-floating population samples, and checked that the extracted samples still meet the balanced distribution of the original place. (The sample numbers corresponding to inter-provincial migration, inter-city migration within a province and inter-district migration within a city are less than 3.5 times the number of the non-floating population, so all of the samples of the three patterns are used.) Both OLS and PSM were used to estimate the migration effects of the four flowing patterns. The results are shown in Table 8.

**Table 8:** The migration effect of the four patterns floating population-OLS and PSM.

Pattern	PSM				OLS	
	ATT ^1^	t-value ^2^	Sample		the coefficient of the migration	t-value
			untreated	treated		
*flowing into the three largest megacities*	0.4496-0.592	14.96	8761	30404	0.4956	58.17
*inter-provincial migration*	0.2306-0.2618	4.42	8761	20599	0.1614	13.91
***inter-city migration within a province***	0.1089-0.1508	2.43	8761	21686	0.0584	6.27
***inter-district migration within a city***	0.0594-0.0879	1.36	8761	18008	-0.0099	-0.95

Note: The bolder and italic ones mean that the regressions of inter-city migration within a province and inter-district migration within a city don’t include the variable “education funding from the government of original place for every student”, because each province is taken as the unit to collect education funding data.

^1^ The ATTs’ intervals of the PSM are estimated using one-to-one, one-to-two, one-to-four, one-to-seven, and one-to-eleven nearest neighbor matching (with replacement).

^2^ The t-value of the PSM is the median of the t-values for the different matching parameters of nearest neighbor matching.

The migration effects of the four different flowing scales were estimated using OLS and PSM (using various matching parameters for nearest neighbor matching). The estimation results of the PSM show that migration could increase wages by 44.96% to 59.20%, 23.06% to 26.18%, 10.89% to 15.08%, and 5.94% to 8.79% respectively for the four migration patterns. We also find that the migration effects are robust for the first three patterns because of the small interval range of ATTs and the sufficiently large t-values for all choices of matching parameters. For the pattern of inter-district migration within a city, although the interval range of ATT is small enough, the t-value of the ATT is less than the boundary value of 1.96, so inter-district migration within a city has no significant impact on wage. From the t-values in the seventh column of Table 8, we similarly see that the migration effects which are estimated by OLS are significant except in the case of inter-district migration within a city. By comparing the results of PSM and OLS in Table 8, the migration effects estimated by OLS were less than those of PSM for each flowing scale as a whole. Meanwhile, both the results of OLS and PSM indicate the following relationship: the migration effect of flowing into the three largest megacities > the effect of inter-provincial migration > the effect of inter-city migration within a province.

#### The migration effects of the four different flowing scales: Three China’s northeastern provinces

The economic downturn of the three northeastern provinces made the outflow of the population very significant in the past few years. The Report of China’s Floating Population made by the NHFPCC in 2015 shows that the population of the three northeastern provinces had a net outflow of about 180,000 in the past 10 years, and according to the sixth census in 2010 and the fifth census in 2000 most of those who have left were young people. According to the statistics, the outflow of the northeastern population was mainly towards the Pan-pearl River Delta, Yangtze River Delta and Beijing-Tianjin-Hebei Metropolis Circle, which are the three fastest economic growth regions. Therefore, it is important to estimate the wage difference before and after the migration for the population of China’s three northeastern provinces to find some deep reasons for the phenomenon of net outflow.

We extracted the samples of floating and non-floating populations of the three northeastern provinces from the 2015 NHFPCC dataset according to the three original provinces: Liaoning, Jilin, and Heilongjiang. Finally, the total number of samples that were extracted was about 13,473, including a floating population of 12,469 (including the flowing into the three largest megacities, inter-provincial migration, inter-city migration within a province, and inter-district migration within a city; the numbers of the four patterns were 882, 4605, 5047, and 1935 respectively.) and a non-floating population of 1,004. As above, both OLS and PSM were used to estimate the migration effects of the four flowing scales for the population of the three northeastern provinces, but because the values of the variable “education funding from the government of original place for every student” are not very different in the three northeastern provinces, and the values were all less than 40,000RMB, this variable will not be included in the model. Meanwhile, the three northeastern provinces had the same variable original place, so the variable original place will also not be included in the model. The estimated results are in Table 9.

**Table 9:** The migration effect of the four patterns in the three northeastern provinces of China-OLS and PSM.

Pattern	PSM				OLS	
	ATT ^1^	t-value ^2^	Sample		the coefficient of the migration	t-value
			untreated	treated		
*flowing into the three largest megacities*	0.6541-0.6839	15.96	1003	882	0.7310	25.61
*inter-provincial migration*	0.2535-0.2905	4.39	1004	4536	0.2221	11.29
*inter-city migration within a province*	0.0512-0.0691	2.05	1004	4975	0.0626	3.34
*inter-district migration within a city*	0.0272-0.0407	1.71	1004	1935	0.0331	1.33

^1^ The ATTs’ intervals of the PSM are estimated using one-to-one, one-to-two, one-to-four, one-to-seven, and one-to-eleven nearest neighbor matching (with replacement).

^2^ The t-value of the PSM is the median of the t-values for the different matching parameters of nearest neighbor matching.

From Table 9, we see that the t-values are larger than the boundary value of 1.96 except for inter-district migration within a city, so the migration effects of the first three patterns estimated by the PSM and OLS are significant. The estimated results of the PSM show that the first three migration patterns could increase wages by 65.41% to 68.39%, 25.35% to 29.05%, and 5.12% to 6.91% respectively. Most importantly, the ATT interval is not very big for any pattern regardless of the parameter values, so the estimated results are sufficiently robust. By comparing the results of PSM and OLS in Table 9, we see that the results of PSM are significantly less than those of OLS for each pattern, so the selection bias is very serious, so it is necessary to use PSM to estimate the migration effect. At the same time, the results of OLS and PSM also indicated the same relationship: the effect of flowing into the three largest megacities > the effect of inter-provincial migration > the effect of inter-city migration within a province.

In summary, in order to get the migration effects for different flowing scales, the 2015 NHFPCC dataset were divided into four patterns according to the different flowing scales, and then used with PSM to estimate the wage difference before and after migration. Meanwhile, the floating and non-floating population samples of China’s three northeastern provinces were extracted from the whole dataset, and used to estimate the migration effect of three northeastern provinces. For the overall samples, the results show that the migration could increase wages by 44.96% to 59.20%, 23.06% to 26.18%, and 10.89% to 15.08% respectively for the first three patterns, so the people who flowed into the three largest megacities could earn more about 35% and 45% respectively compared to those in the other two patterns. For China’s three northeastern provinces, the first three patterns could increase wages by 65.41% to 68.39%, 25.35% to 29.05%, and 5.12% to 6.91%, meanwhile the people who flowed into the three largest megacities earned more nearly 40% and 63% respectively compared to those in the other two patterns.

#### Discussion

Recently, some scholars of China have proposed views about local employment and local urbanization near the floating population’s place of origin. Meanwhile, some local governments (except for the three largest megacities) actively create policies designed to attract talent and encourage the floating population to settle down. Combining the empirical analysis of the migration effects of the different floating scales above, the views of local employment and local urbanization near one’s place of origin seem difficult to implement in reality, and the effects of the relevant policies seem similarly to be relatively poor. The local employment and local urbanization policies are only applicable to a few economic developed areas where natives are difficult to get a higher wage by flowing to other cities. It would therefore appear that the local employment and local urbanization nearby the original places cannot be widely applied in China.

On the basis of the empirical analysis, we can make a couple of inferences. Since the migration effect of flowing into the three largest megacities is very significant, the number of the floating population in the three largest megacities will likely increase. In light of this, in order to solve the increasingly serious largest megacity disease affecting e.g. Beijing, it is imperative to transfer Beijing’s non-capital functions to nearby places and remove the floating population as soon afterwards as possible. This is a complex problem, and any solution needs to take into account the interests of the various parties involved.

For the three largest megacities, the removal of the floating population would require a series of steps. First of all, the universities, public institutions, state-owned enterprises would need to be transferred into other cities; Second, the industries in which the floating population has been gathering should be similarly moved, including the wholesale clothing market, the small commodity market, the building material market, and so on. Cities accepting the non-orientation functions of the three largest megacities should adapt themselves to their new functions and therefore improve the migration effects of the inter-provincial migration and inter-city migration within a province for the floating population. Additionally, the cities that accept these functions should construct the necessary industries, establish the relevant policies, and create a fair competitive environment to support the new functions. They will also need to increase job opportunities, and improve regional economic vitality, improve the sense of identity of the floating population who flow along with the new functions in order to attract more floating populations. Moreover, the floating population in the largest megacities will need to catch the trend to develop business in the new cities which will have good policies, environments and lower costs of living and business. It may produce a high utility in terms of net income and happiness level.

## Conclusion

The study estimates the migration effect of China’s floating population mainly using the PSM method based on the 2015 NHFPCC Migrants Population Dynamic Monitoring Survey Data.

In a certain sense, the PSM method can be regarded as a kind of re-sampling, which tries to make the data of the treatment group as close as possible to that of the untreated group using re-sampling matching. It is therefore a very popular method of empirical analysis, especially in the medical studies [45–48]. However, the PSM method also has its limitations. First, the PSM method requires a relatively large sample size to achieve a high-quality matching; Second, the propensity scores of the treatment group and the untreated group require a large common support range; Third, the PSM method only estimates the propensity scores of the measurable variables, so there will still be some hidden biases in the presence of non-measurable variables; and so on.

By estimating the migration effect of China’s floating population by using the 2015 NHFPCC survey data, we get two main conclusions:

First, there is a significant and positive effect on the average wage growth for the overall floating population of China, especially those who flowed into the three largest megacities. This is the main reason why the floating population would like to flow into the three largest megacities. There are additionally more job opportunities and richer work types in the three largest megacities, so the floating population will find it easier to obtain a suitable job.

Second, the migration effects for the floating population of the three northeastern provinces are significantly higher than the national average in these two patterns of flowing into the three largest megacities and inter-provincial migration, but there is almost no change in wage for the inter-city migration within a province. This indicates that the wage level of China’s three northeastern provinces is relatively low, so there will be a large increase in wage after migration; and the main reason why there is no significant effect on the inter-city migration within a province may simply be that the wage level in the three northeastern provinces is generally quite low. In general, the low wage level indicates the reality that the economic development of the three northeastern provinces has seriously lagged, job opportunities are relatively scarce, and labor is relatively abundant. Indeed, this has been the overall situation of the three northeastern provinces in recent years.

There are many directions for future research. First, given the large nominal wage gap among regions, the first instinct of the people is to flow in order to get higher salaries, but this usually does not take into account the higher cost of living, which could be used to estimate the net income change after migration. Second, as a result of China’s household registration policy, migration will increase healthcare costs for the floating population and education costs for their children in new cities, because welfare for public healthcare and education can now in China only be gotten in one’s city of household registration. Thus, changes in the healthcare and education costs also are good directions to study the migration effect, and need be appropriately considered in the study of net income change before and after migration. Third, the migration effects on non-wage benefits such as cultural amenities, the cost of finding a job and environmental quality are also good research areas. Fourth, studying which kind of migration makes it easiest to settle down in one’s destination is also a good direction. Fifth, one could try to estimate the effect of the Belt and Road using the PSM method.

## References

[1] Barkley A P. The determinants of the migration of labor out of agriculture in the United States, 1940–85. American Journal of Agricultural Economics, 1990, 72(3):567–573. 10.2307/1243025

[2] Greenwood M J. Internal migration in developed countries Handbook of Population & Family Economics, 1997, 1, part b(97):647–720.

[3] Chiswick B R. Are Immigrants Favorably Self-Selected? American Economic Review, 1999, 89(2):181–185. 10.1257/aer.89.2.181

[4] Shen J. Changing Patterns and Determinants of Interprovincial Migration in China 1985–2000. Population Space & Place, 2012, 18(3):384–402. 10.1002/psp.668

[5] Li H, Zahniser S. The Determinants of Temporary Rural-to-Urban Migration in China. Urban Studies, 2002, 39(12):2219–2235. 10.1080/0042098022000033836

[6] Chen A, Coulson N E. Determinants of Urban Migration: Evidence from Chinese Cities. Urban Studies, 2002, 39(12):2189–2197. 10.1080/0042098022000033818

[7] Polachek S W, Horvath F W. A life cycle approach to migration: analysis of the perspicacious peregrinator. Research in Labor Economics, 1977, 35:349–395. 10.1108/S0147-9121(2012)0000035037

[8] Plane D A. Demographic Influences on Migration. Reg Stud, 1993, 27(4):375–383. 10.1080/00343409312331347635 12344801

[9] Zaiceva A, Zimmermann K F. Scale, diversity, and determinants of labour migration in Europe. Oxford Review of Economic Policy, 2008, 24(24):428–452.

[10] Ham J C, Reagan P B, Li X. Propensity Score Matching, a Distance-Based Measure of Migration, and the Wage Growth of Young Men. Staff Reports, 2005.

[11] Harris J R, Todaro M P. Migration, Unemployment and Development: A Two-Sector Analysis. American Economic Review, 1970, 60(1):126–142.

[12] Wang J Y, Yan-Xia G E, Zeng J. Analysis on Floating Population’s Long-term Residence Willingness in the Rapid Growth City—A Case Study of Ningbo City. Journal of Hebei University (Philosophy and Social Science), 2012, 1: 021.

[13] Gao W, Smyth R. What keeps China’s migrant workers going? Expectations and happiness among China’s floating population. Journal of the Asia Pacific Economy, 2011, 16(2):163–182. 10.1080/13547860.2011.564749

[14] Luo E L. Influence on the willingness to live in cities by employability of migrant workers: taking Shanghai City for example. Urban Problems, 2012, 149(1):21–27.

[15] Su Q, Zhou C. Non-farming Employment of Rural Women in Towns and Their Willingness to Settle. Problem of Agricultural Economy, 2005, 5: 007.

[16] Wei Z X. A Region-specific Comparative Study of Factors Influencing the Residing Preference among Migrant Population in Different Areas: Based on the Dynamic Monitoring & Survey Data on the Migrant Population in Five Cities of China. Population & Economics, 2013, 4: 12–20.

[17] Meng Z M, Wu R J. A Research on Living Intentions of Floating Population. Population & Development, 2011, 17(3): 11–18.

[18] Rozelle S, Taylor J E, Debrauw A. Migration, Remittances, and Agricultural Productivity in China. American Economic Review, 1999, 89(2):287–291. 10.1257/aer.89.2.287

[19] Altonji J G, Card D. The effects of immigration on the labor market outcomes of less-skilled natives In immigration, trade, and the labor market. University of Chicago Press, 1991: 201–234.

[20] Fields G S. Rural-urban migration, urban unemployment and underemployment, and job-search activity in LDCs. Journal of Development Economics, 1975, 2(2):165–187. 10.1016/0304-3878(75)90014-0 12265914

[21] Borjas G J. The impact of immigrants on the earnings of the native-born. Immigration Issues & Policies, 1985.

[22] Withers G. Migration and the labour market: Australian analysis. 1986.

[23] Pope D, Withers G. Do migrants rob jobs? Lessons of Australian history, 1861–1991. The Journal of Economic History, 1993, 53(4): 719–742. 10.1017/S0022050700051299

[24] Addison T, Worswick C. The Impact of Immigration on the Earnings of Natives: Evidence from Australian Micro Data. Economic Record, 2002, 78(240):68–78. 10.1111/1475-4932.00040

[25] Butcher K F, Card D. Immigration and Wages: Evidence From the 1980’s. American Economic Review, 1991, 81(2):292–296.

[26] Greenwood M J, Mcdowell J M. The Factor Market Consequences of U.S. Immigration. Journal of Economic Literature, 1986, 24(4):1738–1772.

[27] Borjas G J. Friends or strangers: the impact of immigrants on the U.S. economy. New York New York Basic Books, 1993(4):731–732.

[28] Shaw K L. The influence of human capital investment on migration and industry change. Journal of Regional Science, 1991, 31(4): 397–416. 10.1111/j.1467-9787.1991.tb00157.x

[29] Bartel A P. The migration decision: What role does job mobility play? The American Economic Review, 1979, 69(5): 775–786.

[30] Hunt J C, Kau J B. Migration and wage growth: a human capital approach. Southern Economic Journal, 1985, 51(3):697–710. 10.2307/1057873 12279899

[31] Gabriel P E, Schmitz S. Favorable Self-Selection and the Internal Migration of Young White Males in the United States. Journal of Human Resources, 1995, 30(3):460–471. 10.2307/146031

[32] Yankow J J. Migration, Job Change, and Wage Growth: A New Perspective on the Pecuniary Return to Geographic Mobility. Journal of Regional Science, 2003, 43(3):483–516. 10.1111/1467-9787.00308

[33] Tunali I. Rationality of migration. International Economic Review, 2000, 41(4): 893–920. 10.1111/1468-2354.00089

[34] Stark O, Bloom D E. The New Economics of Labor Migration. American Economic Review, 1985, 75(2):173–178.

[35] Lewis W A. Economic Development with Unlimited Supplies of Labour. Manchester School, 1954, 22(2):139–191. 10.1111/j.1467-9957.1954.tb00021.x

[36] Nakosteen R A, Zimmer M A. Migration and income: the question of self-selection. Southern Economic Journal, 1980: 840–851. 10.2307/1057152 12310727

[37] Nakosteen R A, Zimmer M A. The effects on earnings of interregional and interindustry migration. Journal of Regional Science, 1982, 22(3): 325–341. 10.1111/j.1467-9787.1982.tb00756.x 12338825

[38] Robinson C, Tomes N. Self-Selection and Interprovincial Migration in Canada. Canadian Journal of Economics, 1982, 15(3):474–502. 10.2307/134762

[39] Rosenbaum P R, Rubin D B. The Central Role of the Propensity Score in Observational Studies for Causal Effects. Biometrika, 1983, 70(1):41–55. 10.1093/biomet/70.1.41

[40] Sobel M E. Causal Inference in the Social and Behavioral Sciences Handbook of Statistical Modeling for the Social and Behavioral Sciences. Springer US, 1995:1–38.

[41] Winship C, Sobel M, Hardy M, Bryman A. Causal Inference in Sociological Studies Handbook of Data Analysis, 2004.

[42] Winship C, Morgan S L. The Estimation of Causal Effects from Observational Data. Annual Review of Sociology, 1999, 25(1):659–706. 10.1146/annurev.soc.25.1.659

[43] Bryson A, Dorsett R, Purdon S. The Use Of Propensity Score Matching In The Evaluation Of Active Labour Market Policies. Lse Research Online Documents on Economics, 2002.

[44] Caliendo M, Kopeinig S. Some practical guidance for the implementation of propensity score matching. Journal of economic surveys, 2008, 22(1): 31–72. 10.1111/j.1467-6419.2007.00527.x

[45] Tao B, Pietropaolo M, Atkinson M, Schatz D, Taylor D. Estimating the Cost of Type 1 Diabetes in the U.S.: A Propensity Score Matching Method. PLoS One, 2010, 5(7): e11501 10.1371/journal.pone.0011501 20634976 PMC2901386

[46] Hu H, Duan Z, Long X, Hertzanu Y, Shi H, Liu S, et al Sorafenib Combined with Transarterial Chemoembolization versus Transarterial Chemoembolization Alone for Advanced-Stage Hepatocellular Carcinoma: A Propensity Score Matching Study. PloS one, 2014, 9(5): e96620 10.1371/journal.pone.0096620 24817002 PMC4016022

[47] Huang Y P, Chen L S, Yen M F, Fann C Y, Chiu Y H, Chen H H, et al Parkinson’s Disease Is Related to an Increased Risk of Ischemic Stroke—A Population-Based Propensity Score-Matched Follow-Up Study. PLoS One, 2013, 8(9): e68314 10.1371/journal.pone.0068314 24023710 PMC3759416

[48] Huang T S, Huang S S, Shyu Y C, Lee C H, Jwo S C, Chen P J, et al A Procalcitonin-Based Algorithm to Guide Antibiotic Therapy in Secondary Peritonitis following Emergency Surgery: A Prospective Study with Propensity Score Matching Analysis. PloS one, 2014, 9(3): e90539 10.1371/journal.pone.0090539 24594916 PMC3942439

